# Dynamic Fall Recovery Control for Legged Robots via Reinforcement Learning

**DOI:** 10.3390/biomimetics9040193

**Published:** 2024-03-22

**Authors:** Sicen Li, Yiming Pang, Panju Bai, Shihao Hu, Liquan Wang, Gang Wang

**Affiliations:** 1 College of Mechanical and Electrical Engineering, Harbin Engineering University, Harbin 150001, China; ihuhuhu@hrbeu.edu.cn (S.L.); pangyiming@hrbeu.edu.cn (Y.P.); baipanju@hrbeu.edu.cn (P.B.); jhjsb@hrbeu.edu.cn (S.H.); wangliquan@hrbeu.edu.cn (L.W.); 2 National Key Laboratory of Autonomous Marine Vehicle Technology, Harbin 150001, China; 3 College of Shipbuilding Engineering, Harbin Engineering University, Harbin 150001, China

**Keywords:** legged robots, fall recovery, reinforcement learning

## Abstract

Falling is inevitable for legged robots when deployed in unstructured and unpredictable real-world scenarios, such as uneven terrain in the wild. Therefore, to recover dynamically from a fall without unintended termination of locomotion, the robot must possess the complex motor skills required for recovery maneuvers. However, this is exceptionally challenging for existing methods, since it involves multiple unspecified internal and external contacts. To go beyond the limitation of existing methods, we introduced a novel deep reinforcement learning framework to train a learning-based state estimator and a proprioceptive history policy for dynamic fall recovery under external disturbances. The proposed learning-based framework applies to different fall cases indoors and outdoors. Furthermore, we show that the learned fall recovery policies are hardware-feasible and can be implemented on real robots. The performance of the proposed approach is evaluated with extensive trials using a quadruped robot, which shows good effectiveness in recovering the robot after a fall on flat surfaces and grassland.

## 1. Introduction

Legged robots are capable of reliably traversing challenging uneven terrains beyond the reach of classical wheeled mobile robots [1,2,3,4]. However, designing controllers that are robust enough to cope with this diversity has been a long-standing challenge in robotics.

When a robot performs tasks in a work environment, it inevitably encounters external disturbances, such as pushing, collisions, and load changes. These external disturbances can cause the robot to deviate from its intended trajectory or fall. If the legged robot falls and is unable to return to an upright position autonomously, a human operator must intervene. Therefore, having the ability to dynamic recovery from a fall is a valuable skill for maintaining stable locomotion [5]. Recovery from a fall is exceptionally challenging for existing methods, since it involves multiple unspecified internal and external contacts. It requires fine coordination of actions across all limbs and must use momentum to flip the robot dynamically.

One possible approach is to represent the recovery behavior as a well-tuned joint trajectory that needs to be replayed: this approach is already used in some commercial systems [6]. Such trajectories require laborious manual adjustment. It takes a long time to execute and has a low success rate because the dynamics are not considered in the motion planning or control. The MPC-based methods [7,8] focus on the unified processing of leg motion, which can find reliable motion trajectories in real-time and is robust to external disturbances. These methods typically require assumptions about the robot’s contact state with the ground to simplify the dynamics model. These assumptions are broken after the robot falls unexpectedly, causing the control pipeline to be terminated.

An alternative for obtaining fall recovery motions is model-free reinforcement learning (RL), where an agent interacts with its environment and learns the control policy through trial and error. Deep Reinforcement Learning (DRL) has shown its successful use for learning fall recovery policies in both simulation and real-world robots [9,10]. The significant advantages of using RL are that it requires less prior knowledge from human experts and is less labor intensive compared to manual handcrafting; the trained neural network is a feedback policy that can fast compute actions in real-time, compared to the optimization-based methods [9]. Previous approaches have only demonstrated fall recovery in static states, but there still needs to be more research on fall recovery during dynamic motion. If the robot falls during dynamic motion (such as running), it may cause catastrophic consequences, such as robot damage, if the robot does not respond to the action in time.

To perform dynamic blind running and fall recovery on challenging terrain, the robot state must be estimated. This can be implicitly estimated using trained neural networks and proprioceptive state histories [3,11]. The proprioceptive history helps estimate both the intrinsic robot state and extrinsic environmental variables. However, since the latent variables are not interpretable, they cannot be used with other modules requiring state information. Moreover, encoder training causes substantial computational overhead. An alternative is to estimate the observable state variables [12] directly, but the errors introduced by state estimation may cause the policy to output unreasonable actions.

To address the shortcomings of the existing methods, we present a novel learning framework combining a learning-based state estimation network and a proprioceptive history policy. We trained a dynamic recovery policy using the presented framework and tested it on the real quadruped robot. We hereby report highly dynamic fall recovery locomotion in various indoor and outdoor environments. When a robot falls at speeds of up to 3 m/s, the recovery policy can help the robot regain its balance in less than one second.

This paper makes the following contributions:

(i) We propose a simple end-to-end locomotion learning framework that trains a proprioceptive policy and a learning-based state estimator;
(ii) Using the trained networks, we demonstrate dynamic fall recovery locomotion on terrains;
(iii) We share the training details, such as the domain randomization settings, privileged learning, and network architectures so that other researchers can reproduce our work.

The paper is organized as follows. Section 2 describes related works and their results. Section 3 describes details of our approach. Section 4 presents experimental results and analysis. Finally, Section 5 presents our conclusions.

## 2. Related Works

Fall Recovery Control. Previously, fall recovery controllers for legged robots were heuristically handcrafted to produce trajectories similar to human or animal fall recovery maneuvers, which required extensive manual design [13,14]. Offline planning methods automate the process by predicting fall [15] or calculating trajectories offline based on specific fall postures [16]. This offline planning is not event-based in nature and, therefore, needs more real-time responsiveness to react to external disturbances. Optimization-based methods can compute feasible fall recovery solutions without the need to handcraft trajectories directly [17,18]. Model Predictive Control (MPC) [19] enables a system to make current decisions while considering their impact on the future through predictive modeling, which has shown promise in recent research on legged locomotion. Recently, some complex leg models, such as single rigid-body models [20,21], central dynamics models [22,23], and whole-body models [24], have been used to improve the locomotor skills of legged robots, especially quadruped robots. These MPC-based strategies focus on the unified processing of legged motions and can find reliable motion trajectories in real time, which are robust to external disturbances. This paradigm relies on explicitly specifying contact points manually or automatically. It requires advanced optimization schemes that grow exponentially in computational power and time as complexity increases and are too slow for real-time solutions, making closed-loop control impractical for robotic fall recovery.

Reinforcement Learning for Fall Recovery. Some of the earliest attempts to integrate learning into locomotion can be traced back to the DARPA plan [25,26]. More recently, reinforcement learning has become a popular method for learning locomotion policies for legged robots [27,28,29]. Deep Reinforcement Learning (DRL) becomes attractive for acquiring motor skills: an artificial agent can learn desirable behaviors by rewarding intended outcomes and penalizing undesired ones. Since policies are usually learned in simulations, they are usually difficult to deploy directly on real robots. One approach is to train directly in the real world [30]. However, such policies are limited to simple settings, and scaling to complex settings requires unsafe exploration and a large number of samples. Therefore, additional efforts are usually required to transfer the learned policies to real robots [31], including constructing more accurate simulations [5,29], dynamic randomization [32], motion imitation [33,34], privilege learning [28], and meta-learning [35]. Using policies trained in simulation, ANYmal [5] is capable of precisely and energy-efficiently following high-level body velocity commands, running faster than ever, and recovering from falls even in complex configurations. Ref. [36] demonstrated successful multi-skill locomotion on a real quadruped robot that autonomously performed coherent trotting, steering, and fall recovery. Ref. [9] proposed a design guideline for selecting key states for initialization and showed that the learned fall recovery policies are hardware-feasible and can be implemented on real robots. Furthermore, DRL has been used to learn fall recovery for humanoid character animation in physics simulation [10]. However, learning dynamic fall recovery policies for robots still needs to be solved.

Sim-to-Real Reinforcement Learning. Owing to the limitations of gathering real-world data, i.e., sample inefficiency and the cost of collecting data, simulation environments are utilized for training the different agents [30]. Simulation-based training involves inherent mismatches with real-world settings. To bridge the gap between simulation and reality, additional efforts are usually required to transfer the learned policies to real robots [31]. Domain randomization [30,37] trains policies with a wide range of environment parameters and sensor noises to learn behaviors that are robust in this range. However, domain randomization trades optimality for robustness, leading to an over-conservative policy [38]. Policy distillation is the process of extracting knowledge to train a new network that can maintain a similarly expert level while being significantly smaller and more efficient [39]. Building on such approaches, privileged learning [28,40] first trains a teacher policy with privileged information and then distills the teacher policy into a student policy via supervised learning. While practical implementations show the efficiency of the different methods, wider theoretical and empirical studies are required to understand the effect of these techniques in the learning process better.

## 3. Method

We aim to develop an RL-based control framework that can help a robot recover from a fall when it is subjected to an external disturbance and loses its balance. We assume the robot has an Inertial Measurement Unit (IMU) and joint encoders.

### 3.1. Overview

**Figure 1:**
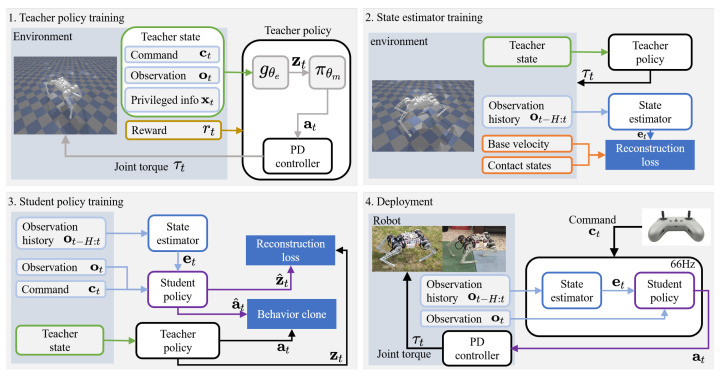
Overview.

An overview of our control framework is illustrated in Figure 1. We train a neural network policy and a state estimator in simulation and then perform zero-shot sim-to-real transfer. Due to the sparsity of rewards, agents are usually unable to learn dynamic fall recovery policies within a reasonable time budget, and thus, learning control policies through reinforcement learning alone has proven unsuccessful in learning controllers for legged robots. To address this problem, a teacher–student training paradigm, also known as privileged learning, is an implicit system identification method [28] that can be used.

Although implicit identification methods can mitigate some of the problems associated with partial observation to some extent, due to the lack of state information, the privileged learning and the reward term alone cannot teach good recovery policies. Our solution to this problem is introducing a state estimation network that provides informative state estimates online. Our method consists of four stages, as described below.

First, a teacher policy is trained with RL over randomly generated terrain with random disturbances. The policy can access privileged information such as base velocities, ground friction, and the introduced disturbances.

Second, a state estimator that outputs current robot state variables, such as foot contact state and body speed, is trained using supervised learning. Training samples are collected with the teacher policy.

Third, a student policy is trained to replicate the teacher policy’s actions. The teacher policy guides the learning of the student controller via behavioral cloning. The student controller solely uses sensors available on the real robot [3] and state variables estimated by the state estimator. The policy inputs robot proprioceptive observations and estimated states and outputs joint position commands that are then converted into joint torques by a proportional-derivative (PD) controller.

Finally, we transfer the learned student policy and the state estimator to the physical robot and deploy it in the real world with onboard sensors without additional training.

### 3.2. Problem Formulation

We formulate our control problem in discrete time dynamics [41], with states s, actions a, reward r(s,a), and dynamics $p(s^{\prime }|s,a)$. The discounted return $R=\sum_{k=0}^{\infty }\gamma^{k}r_{k}$ is the total accumulated rewards from timestep *t*, γ∈[0,1] is a discount factor determining the priority of short-term rewards. The objective is to find the optimal policy $\pi_{\phi }\left(s|a\right)$ with parameters ϕ, which maximizes the expected return $J\left(\phi \right)=E_{p_{\pi }}\left[R_{t}\right]$.

When all states are observable such that $o_{t}=s_{t}$, this can be considered a Markov Decision Process (MDP) [42,43]. When there is unobservable information, such as external forces or full terrain information in our case, the dynamics are modeled as a Partially Observable Markov Decision Process (POMDP) [41,44]. In the quadrupedal locomotion task, there is unobservable information, such as external forces or full terrain information, and the dynamics are modeled as a POMDP [41].

The POMDP can be reconstructed as an MDP by defining *n*-size observations as state [12]:

(1) $$\begin{array}{c} s_{t}\overset{\mathrm{def}}{=}\left(o_{t-H},a_{t-H-1},o_{t-H+1},a_{t-H},\cdots ,o_{t},a_{t-1}\right), \end{array}$$

In deep reinforcement learning, this is frequently achieved by stacking a sequence of previous observations [45] or by using architectures which can compress past information, such as Recurrent Neural Networks (RNNs) [46] or Temporal Convolutional Networks (TCNs) [47]. In this paper, we use TCNs to convert POMDP to MDP, and thus, apply the RL algorithm.

### 3.3. Simulation Environments

We use pybullet [48] as our simulator to build the training environment. We implemented a system for procedural terrain generation to obtain a diverse set of trajectories with variations in contact positions and orientations. The elevation data are represented as a triangulated grid [49]. The height fields were scaled down always to have a random height between −3cm and 3cm. Slopes are simulated by modifying the direction of gravity.

In some studies, it is possible to terminate the current episode (episodes) immediately after the robot falls or topples over, giving the agent a large negative reward. However, this would not allow the agent to understand the environmental situation after the fall and learn strategies to recover dynamically. Therefore, in this study, episodes are never terminated. The robot is reset to the map origin only when the simulation exceeds a preset time threshold or when the robot moves to the edge of the ground.

### 3.4. Notations

Let us denote the linear velocity in the robot’s base frame as $v_{t}\in R^{3}$, joint torques as $\tau_{t}\in R^{12}$, the position in the world frame as $p_{t}\in R^{3}$, the acceleration of the base in the robot’s base frame ${\ddot{a}}_{t}\in R^{3}$, the velocity of foot as $v_{t}^{f}\in R^{4}$, the binary foot contact indicator vector as $f^{t}\in R^{4}$, and the total power of the robot at the current time step as $W_{t}$. $g_{t}^{\mathrm{ori}}\in R^{3}$ and $\omega_{t}^{\mathrm{ori}}\in R^{3}$ denote the orientation and angular velocities are measured using the IMU. The body orientation command $c_{t}$ is specified by a human operator via remote control. $q_{t}\in R^{12}$ and ${\dot{q}}_{t}\in R^{12}$ are joint angles and velocities.

### 3.5. Reward

The reward function is motivated by previous works [11,12,50]. The reward function encourages the agent to stand without falling and penalizes it for jerky and inefficient motions. The reward function has task reward terms for velocity penalty, as well as a set of auxiliary terms for stability (height constraint and angle velocity penalties on body roll, pitch), safety (penalty on self-collision), smoothness (foot slip, joint torque, and base acceleration penalties), and energy (power of current time step). The smoothness, safety, and energy reward encourage agents to learn stable and natural gaits. The reward at time *t* is defined as the sum of the following quantities:

- Velocity Penalty: $-{∥v_{t}∥}^{2}$;
- Orientation Tracking: $-{∥c_{t}-g_{t}^{\mathrm{ori}}∥}^{2}$;
- Height Constraint: $-|p_{t}^{z}-p_{t}^{\mathrm{target}}|$;
- Angle Velocity Penalty: $-{∥\omega_{t}^{\mathrm{ori}}∥}^{2}$;
- Self-collision Penalty: $-1_{\mathrm{selfcollision}}$;
- Joint Torque Penalty: $-{∥\tau_{t}∥}^{2}$;
- Base Acceleration Penalty: $-{∥{\ddot{a}}_{t}∥}^{2}$;
- Energy: $-W_{t}$;
- Foot Slip: $-{∥\mathrm{diag}\left(f^{t}\right)\cdot v_{t}^{f}∥}^{2}$.

The scaling factor of each reward term is 3.0, 1.0, 10, 0.21, 1.3, 0.018, 0.1, 0.012, and 0.3, respectively.

### 3.6. Domain Randomization

Because of the inconsistency in the physical dynamics between the simulated and real environments, the reality gap often arises when transferring the policy learned in simulation to a real robot [50]. To bridge the reality gap, we randomize the dynamics parameters of the robot. In addition, external force and torque are applied to the robot’s body, and the feet’s friction coefficients are occasionally set to a low value to introduce slippage [28,29]. At the beginning of each training episode, we randomly sample a set of physical parameters and use them in the simulation. The complete list of randomized parameters and their randomization ranges is summarized in Table 1.

Since domain randomization trades optimality for robustness [29], the parameters and their ranges in Table 1 must be chosen carefully to prevent learning an overly conservative running gait. Mass and motor friction are commonly used randomization parameters [27]. In this study, robot mass and joint friction were measured during the design of the robot, thus giving conservative ranges but less certainty for inertia as estimated using Solidworks. In addition, some of the dynamic variables change over time. For example, motor friction will change due to wear. Control frequency and latency will fluctuate due to the nonreal-time nature of the system. The battery voltage will vary depending on whether it is fully charged or not. For these reasons, the choice was made to randomize these parameters and their ranges based on actual measurements plus a small safety factor. The coefficient of friction was randomly sampled between 0.4 and 1.25, as this is the typical range of friction coefficients between the robot’s rubber feet and various terrains. A small amount of noise was also added to the simulated IMU readings since real IMU feedback is typically biased and noisy.

We apply external force and torque to the robot’s body, introduce slippage by setting the friction coefficients of the feet to a low value, and randomize the robot’s dynamics parameters [28,29]. Before each training episode, we randomly select a set of physical parameters for the simulation. The complete list of these randomized parameters and their randomization ranges is found in Table 1.

### 3.7. Teacher Policy

Policies can output low-impedance joint position commands, which are very effective in many tasks. Such controllers outperform torque controllers regarding training speed and final control performance [51]. Although there is always a bi-directional mapping relationship between them, the smoothness of the two action parameterizations is different, and therefore, the training difficulty is different. In addition, the positional strategy has an advantage in training because it starts as a stable controller. Individual joints do not easily swing to the limit position, whereas the torque controller can easily cause the joints to get stuck in the limit position at first. Therefore, we designed the action space of the strategy network as an impedance controller (PD controller).

The action $a_{t}$ is a 12-dimensional vector with desired joint positions. A PD controler is used to calculate torque $\tau =Kp\left(\hat{q}-q\right)+Kd\left(\hat{\dot{q}}-\dot{q}\right)$. Kp and Kd are manually specified gains, and the target joint velocities $\hat{\dot{q}}$ are set to 0. The proportional gain is 30Nm/rad, and the derivative gain is 0.5Nm/rad·s. We chose these low gains to promote smooth motions and did not tune them during our experiments [11]. Kp and Kd were manually specified gains. These lower Kp were chosen to promote smooth motion, but they were not adjusted during the experiment. Based on practical experience, small changes in Kp can ensure the final motion performance. For example, increasing the Kp to 35Nm/rad does not significantly change the performance. In reinforcement learning, the policy is trained to predict the occurrence of positional errors and can even utilize them to generate acceleration and interaction forces. Furthermore, due to the dynamics randomization technique, the trained control strategy does not depend entirely on the dynamics: the intelligence inevitably learns to apply an appropriate impulse of motion to the environment. In conventional control, impulse-based [52] control methods are more robust to system variations and model inaccuracies.

The robot’s sensors provide joint angles $q_{t}\in R^{12}$ and joint velocities ${\dot{q}}_{t}\in R^{12}$, measured using motor encoders, and $g_{t}^{\mathrm{ori}}\in R^{3}$, $\omega_{t}^{\mathrm{ori}}\in R^{3}$, which denote the angular velocities and the orientation and are measured using the IMU. Policy π takes as input a history of previous observations and actions denoted by $o_{t-H:t}$, where $o_{t}=\left[q_{t},{\dot{q}}_{t},g_{t}^{\mathrm{ori}},\omega_{t}^{\mathrm{ori}},a_{t-1}\right]$. Because we are learning a command-conditioned policy, the input to the policy is $o_{t}⨁c_{t}$, where ⨁ denotes the concatenation operation. During deployment, the command $c_{t}=\left\{v_{t}^{cmd},d_{t}^{cmd}\right\}$ is specified by a human operator via remote control.

The teacher observation (see Table 2) is defined as $s_{t}^{teacher}=\left(o_{t-H:t},x_{t-H:t},c_{t}\right)$, where H=4. $x_{t}$ is the privileged state. The privileged state $x_{t}$ includes contact states, contact forces, friction coefficient, external forces and torques applied to the body, and swing phase duration. The teacher policy $\pi_{\theta }^{teacher}$ consists of two multilayer perception (MLP) components: a state encoder $g_{\theta_{e}}$ and $\pi_{\theta_{m}}$, such that $a_{t}=\pi_{\theta_{m}}\left(z_{t}\right)$ where $z_{t}=g_{\theta_{e}}\left(s_{t}\right)$ is a latent representation. We optimize the teacher parameters together using PPO [53]. The PPO training parameters used for all experiments are provided in Table 3.

Privileged learning training enables the agent to specialize its behavior to the current dynamics instead of learning a single behavior that works across different dynamics. This so-called implicit system identification approach has been previously developed in several works involving object re-orientation with a multi-finger hand [54], self-driving cars [55], and locomotion [27]. However, this recognition capability is still limited, and recognizing the robot’s state characteristics in the event of a fall is very difficult. The state estimator is an effective way to address this problem and complement the privileged learning blindness.

**Table 3:** Hyperparameters of PPO.

Hyperparameters	Value
Optimizer	Adam [56]
learning rate	$3\times 10^{-4}$
timesteps per rollout	20
epochs per rollout	5
number of hidden layers	2
number of hidden units per layer	256
Discount (γ)	0.99
Nonlinearity	ReLU
Minibatch size	4096
entropy bonus	0.01
value loss coefficient	1.0
clip range	0.2

### 3.8. State Estimator

The estimator network is designed to predict the state of the robot without utilizing a dedicated estimation algorithm. In this paper, linear velocity and contact probability are estimated. Linear velocity estimation and contact state are essential for dynamic fall recovery control. The implementation of the robot is made simpler by not requiring a complex state estimation algorithm. This has the added benefit that the controller is robust to the inevitable errors of the state estimator. As shown in the paper [57], the linear velocity estimation is susceptible to foot slippage in error-ridden environments.

Our end-to-end neural network structure avoids such a challenge in three ways. Firstly, the estimator network trains in an environment full of randomness. Secondly, the samples are collected by the teacher policy, which is very close to the state experienced by real robots. Finally, the student policy trained using the privileged learning approach can effectively identify the state estimation error and respond appropriately.

The estimator network is a temporal convolutional network (TCN) [47] that receives a sequence of *H* proprioceptive observations $o_{t-H:t}$ as input, where H=100. The architecture is shown in Table 4. The estimator network output $e_{t}=\left\{v_{t}^{e},{\mathrm{diag}}^{e}\left(f^{t}\right)\right\}$. We use the Mean Squared Error (MSE) to compute the gradient of the state estimator. All parameters are updated by the Adam optimizer [56] with a fixed learning rate $3\times 10^{-4}$.

### 3.9. Student Policy

The input to the student policy is $s_{t}^{student}=\left(o_{t},c_{t},e_{t}\right)$. We use the same training environment as for the teacher policy, but add additional noise to the student observation $o_{t}^{noise}=n\left(o_{t}\right)$, where $n(o_{t})$ is a Gaussian noise model applied to the observation. The student network outputs action ${\hat{a}}_{t}$ and latent representation ${\hat{z}}_{t}$. The student policy is trained via supervised learning with the dataset aggregation strategy (DAgger) [58]:

(2) $$\begin{array}{c} L={\left({\hat{a}}_{t}-a_{t}\right)}^{2}+{\left({\hat{z}}_{t}-z_{t}\right)}^{2}. \end{array}$$

The teacher policy computes each visited state’s action vectors and latent representations. These outputs of the teacher policy are used as supervisory signals associated with the corresponding states. Equation (2) encourages students to imitate as much of the teacher’s output as possible in terms of actions and latent representations.

The DAgger algorithm is based on Online Learning, which takes the student policies obtained from behavioral clones and interacts them with the environment continuously to generate new data. On this newly generated data, DAgger requests demonstrations from the teacher policy; then, on the augmented dataset, DAgger will retrain student policies with the behavioral clone and then interact with the environment. This process is repeated over and over again. As a result of data augmentation and environment interaction, the DAgger algorithm greatly reduces the number of unvisited states, thus reducing the error. For student policy, we use a fully connected network with 2 hidden layers and 256 units per layer, with a Rectified Linear Unit in each layer [59]. All the parameters are updated by the Adam optimizer [56] with a fixed learning rate.

### 3.10. Implement Details

We implement all programs with Pytorch [60] and NumPy [61]. Ray[tune] [62] is used to build and run distributed applications. PPO collected 50 million simulated time steps in our experiments using 20 parallel agents for policy training. The student policy training process took 3 h. The process can be completed in under 18 h of wall-clock time using an AMD Ryzen 9 5950X CPU and a single NVIDIA RTX 3090 GPU.

## 4. Results and Analysis

Our robots autonomously coordinate different locomotion patterns in unexpected situations to mitigate disturbances and prevent or recover from malfunctions without direct human intervention. For example, when falling at speeds of up to 3 m/s, the learning recovery controller extends the legs to protect the body, then controls the body posture and returns to running. Alternatively, if the robot loses its balance due to a collision with an obstacle, the learned recovery controller controls the legs to support the ground and recover quickly. In the following, we will show the experimental results of the trained neural networks and present the multi-modal locomotion experiments using our method.

### 4.1. Robot Hardware

In this study, we used a quadrupedal robot equipped with motor encoders to measure motor angles and an IMU to measure the orientation and angular velocity of its body. The robot has four legs with three rotational degrees of freedom on each leg, including 6 degrees of freedom for the floating body, giving the robot a combined total of 18 degrees of freedom with 12 active degrees of freedom. Each active degree of freedom is driven using A1 joint motors, which have a peak torque of 33.5 Nm and a maximum speed of 21 rad/s. Our robot shares identical actuators to the A1 robot (https://www.unitree.com/a1). The robot stands at approximately 0.3 m tall and weighs 15 kg.

An NVIDIA Jetson Orin developer kit is integrated into the robot to provide additional computing power. The Orin developer kit has a 2048-core GPU, implementing CUDA to facilitate neural network inferences. Orin communicates with the microcontroller through RS-485 buses. The microcontroller collects the sensor measurements at each control step and sends them back to Orin. In Orin, neural network policies are entered to determine the actions to be taken. These actions are then transmitted to the microcontroller and executed by the A1 joints. Since Orin is not running a real-time operating system, the control loop operates at a variable control frequency of about 60 to 70 Hz.

### 4.2. Simulation Experiments

**Figure 2:**
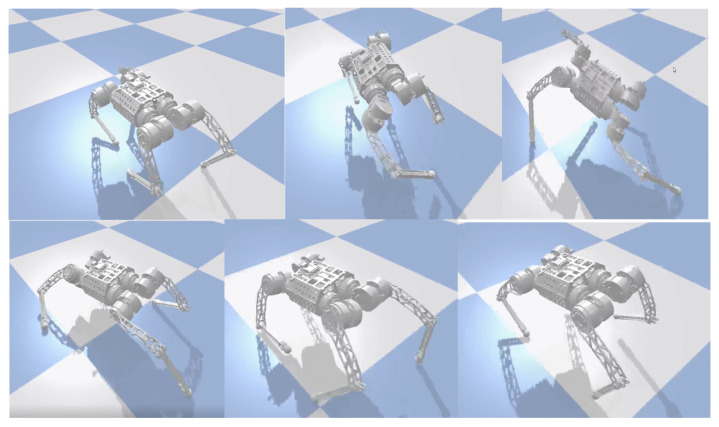
Lateral impact testing in simulation.

**Figure 3:**
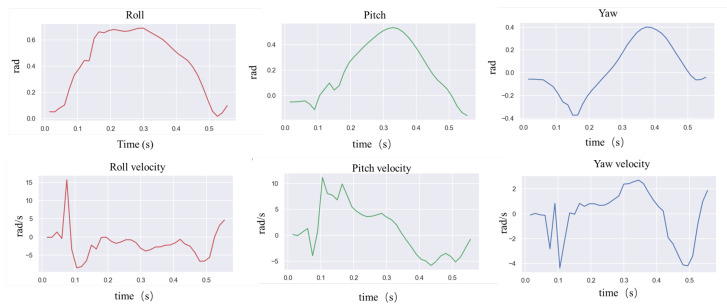
Robot IMU data for lateral impact testing.

In natural environments, robots are easily disturbed by external perturbations or terrain ups and downs, and the robustness of motion control methods needs to be validated. Usually, quadruped robots are more stable in the forward direction than in the measured plane and are more likely to cause a fall when subjected to lateral perturbations. In order to verify the ability of the controller to resist the impact force, a random force of about 300 N is used in the simulation to apply a lateral impact to the robot. The robot walks at a forward speed of 0.8 m/s, and the impact force is applied to the right side of the robot base at a random moment. Figure 2 and Figure 3 shows the results.

**Figure 4:**
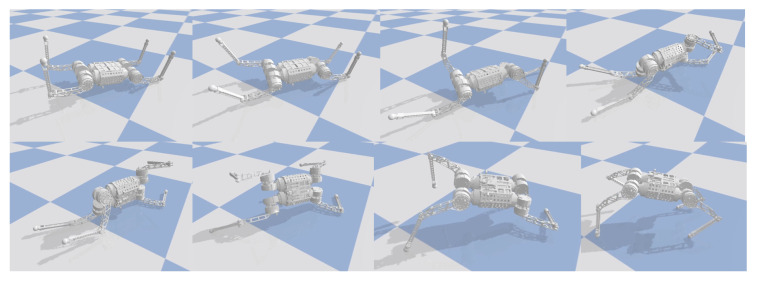
Robotic fall recovery in simulation.

**Figure 5:**
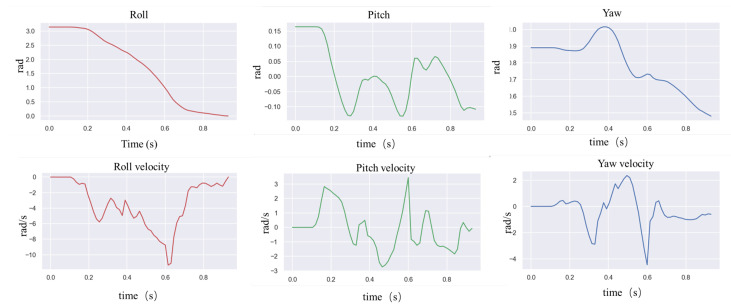
Robot IMU data for fall recovery testing in simulation.

In the simulation, the robot will fall entirely over when it is subjected to an excessive impact. At this point, the robot will extend its legs behind it to support its body, flip it over, and recover from the fall posture. After recovering to the normal posture, the robot resumes standing and continues to move forward. This indicates that the proposed controller has a vital recovery function after a fall. As shown in Figure 4, even in the case of a complete fall, the robot can still get up quickly and return to a standing position. During the recovery process (Figure 5), the robot’s body flipped at a maximum rotation speed of 10 rad/s.

### 4.3. Static Recovery Experiments

When starting the robot from a power-down state, the robot itself is likely to be out of its normal initialization state (e.g., the robot topples over), so the robot needs to have the ability to be able to recover from an arbitrary state to standing. For fall recovery, the DRL agent is rewarded for feedback policies that restore stable postures from various failure states. We applied random initialization to explore diverse robot states and facilitated the agent’s ability to generalize policies for various fall poses.

**Figure 6:**
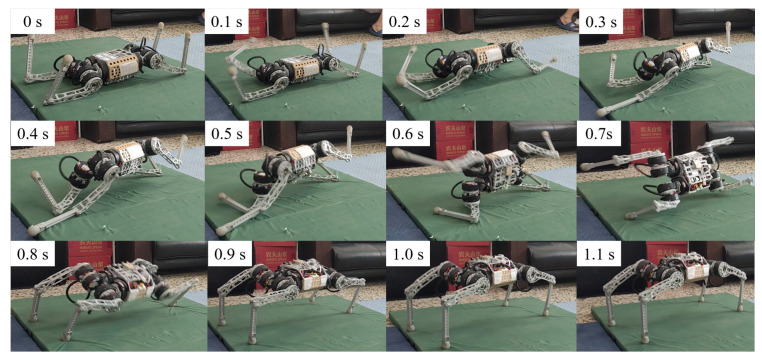
Indoor static recovery results. When the robot is walking, an unknown disruptive force is suddenly given to the robot. The controller quickly adjusts the motion gait and stabilizes the body.

To test whether this controller can recover from a complete tip-over, the robot was first completely powered down and then flipped over 180 degrees and placed on the ground, as shown in Figure 6. After powering up the robot, the controller automatically began to operate. The controller first controls the left front leg and left back leg to contact the ground to support the body flip. After the complete flip, the left front and left back legs were quickly retracted to allow the robot to stand up from the ground using the inertia of the flip. This task is challenging, as the controller must fully recover the robot without historical information.

Upon activation of the robot, the learned control policy quickly controls the control leg to make contact with the ground, thus supporting the body to flip over and resume standing. The results are shown in Figure 6. The entire recovery process lasts approximately one second. We observed that the robot would use the inertia of the tumble to allow the body to regain the desired height more quickly during the tumble. We have conducted several tests in similar scenarios and have a 100% success rate in recovering from falls. The robot flips in a different direction each time it recovers from a fall. This suggests that the neural network controller autonomously chooses the most appropriate recovery direction.

### 4.4. Dynamic Recovery Experiments

**Figure 7:**
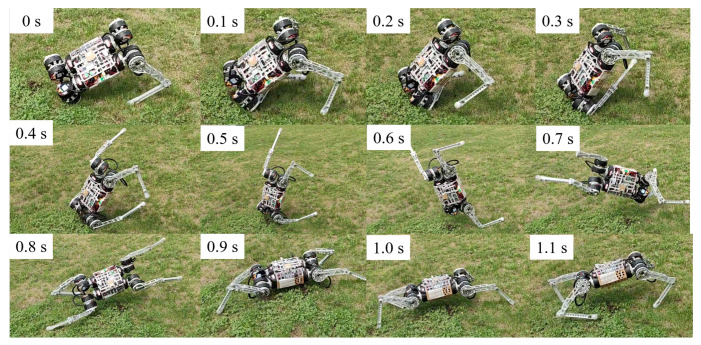
Dynamic recovery experiment result. When the robot falls accidentally while running on grassland, the controller quickly supports the ground so that the robot’s body flips to the correct position and resumes running.

We demonstrate the performance of our controller in challenging outdoor environments, as shown in Figure 7. Traversing these terrains can be challenging for a robot as its feet may sink or stick, causing instability. To maintain stability, the robot must adjust its footholds dynamically, which may lead to periodic instability while walking.

In unexpected situations, our robots autonomously coordinate different locomotion patterns to mitigate disturbances and prevent or recover from malfunctions without human help. Even when falling at speeds of up to 3 m/s, the learning recovery controller extends the legs to protect the body, controls the body posture and returns to running. When the robot collides with an obstacle and falls, the recovery controller controls the legs to support the ground and recover quickly.

These behaviors are remarkably similar to those of biological systems (e.g., cats, dogs, and humans), exhibiting greater versatility and intelligence: the ability to cope with changing and complex situations. In contrast to manually designed fall recovery controllers with fixed patterns, our learning controllers can recover from a wide range of fall situations by responding to dynamic changes through online feedback. In contrast, manual controllers can only respond to a tiny range of situations.

### 4.5. Response to Unexpected Disturbances

**Figure 8:**
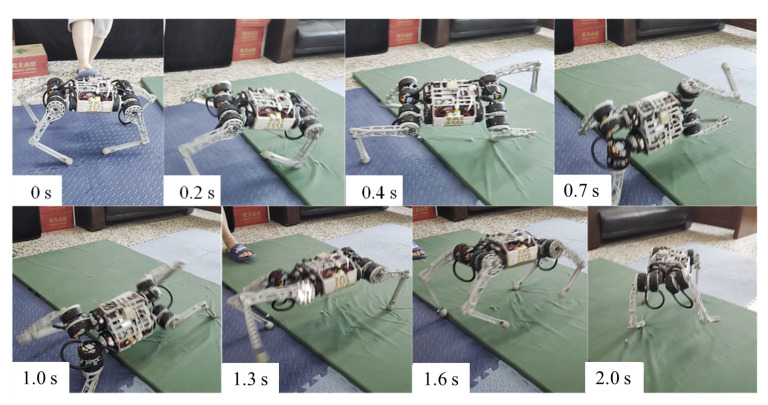
Unexpected disturbances experiment.

**Figure 9:**
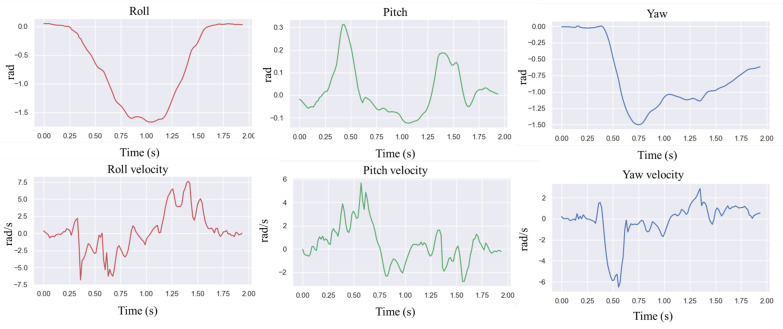
Robot IMU data when applying disruptive force to the robot.

In order to test the robot’s ability to respond to external impacts, a significant impact force was suddenly given from the side of the robot body while the robot was moving forward at 0.8 m/s. Figure 8 above shows how the robot resisted the impact, and Figure 9 shows the IMU data after the impact. The robot loses its balance very quickly after the impact, and when it is about to fall, the controller extends its legs to prevent the body from falling entirely to the ground. From the IMU data, it can be seen that 1 s after the impact, the robot’s roll angle reaches a maximum of −1.5 rad, and the yaw is greatly shifted. Then, the robot quickly used its legs to support the ground, thus recovering its balance. The whole recovery process lasted about 0.5 s, and at this time, the IMU angular velocity reached 7.5 rad/s at maximum.

**Figure 10:**
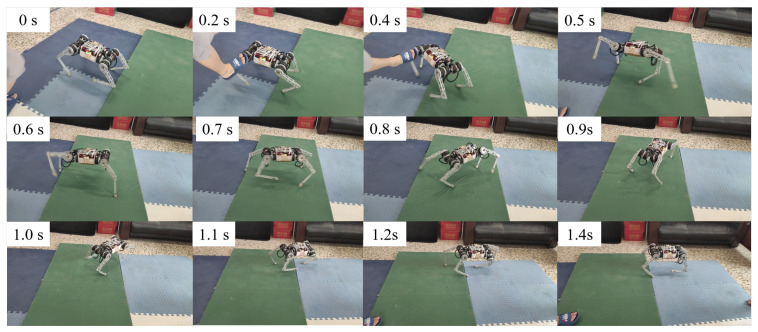
Applying an external disruptive force from behind the robot.

Similar results were obtained by applying the interference force from the side and rear, as shown in Figure 10. The difference is that the robot did not fall completely, but used the support of its legs to reorient the body and mitigate the large inertia caused by the impact. Although the body was deflected by more than 1 rad, it stabilized and returned to normal walking conditions.

We also evaluated the learned locomotion policy through qualitative testing by giving random commands using a joystick. During the experiment, the robot was also subjected to multiple external pushes to the main body. We conducted additional one-hour tests without encountering failures (approximately 30 valid tests), demonstrating the policy’s robustness. It is worth noting that the controllers functioned smoothly in the real world without requiring further adjustments to the physical system.

### 4.6. Analysis

#### 4.6.1. Recovery Strategies

We evaluated the robustness of the recovery policies and classified the learned response behaviors into three strategies. (1) Natural rolling using semi-passive motion (Figure 7). Natural rolling is the behavior of a robot that rolls using inertia and gravity. (2) Active righting and tumbling (Figure 6). Active righting is a strategy in which the robot uses its legs and elbows to propel itself and generate momentum to flip into a prone position. (3) Stride. Figure 8 and Figure 10 represent an example of a stride in which coordination and switching of the support legs are required to restore balance when an external disturbance destabilizes the current motion. This multi-touch switching is naturally achieved through a learning-based strategy.

#### 4.6.2. Cluster Analysis

We analyzed the features learned by the policies separately using t-distributed Stochastic Neighbor Embedding (t-SNE), thus investigating how skills are adapted and distributed in the network. The t-SNE algorithm is a dimensionality reduction technique used to embed high-dimensional data into a low-dimensional space and to visualize them. The 2D projection of the gating network’s activation pattern is obtained with t-SNE, and the neighborhoods and clusters of the samples are visualized. Samples representing similar activation appear nearby, whereas the different ones are distant from each other.

**Figure 11:**
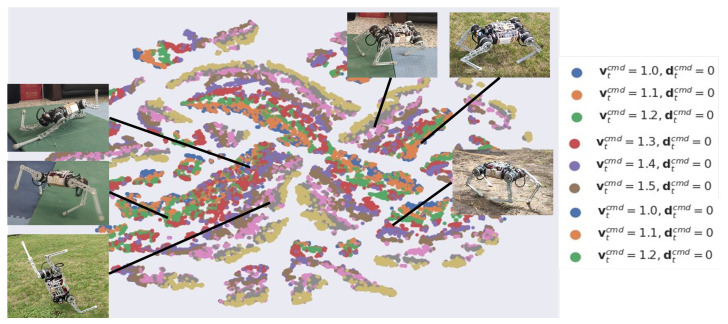
Two-dimensional t-SNE embedding of the representations in the last hidden layer. The color corresponds to the command given to the robot.

In Figure 11, t-SNE analyses of network outputs reveal relationships between motor skills learned by the network. The agent learns unique skills and patterns under various scenarios, revealing diverse skills. Even if the scenarios are very similar, the network reuses specific patterns and features to some extent but also fine-tunes these minor differences.

#### 4.6.3. Comparisons

We compare the performance of our method to baseline methods in simulation:

- Expert Policy. In simulation, we can use the true value of the robot states. This is an upper bound to the performance of our method.
- Concurrent method [12]. Instead of using privileged learning methods, the concurrent method trains both the policy and a neural network state estimator.
- Without state estimator. We can also evaluate the performance of the base policy without the state estimator to ablate the importance of the state estimator.

Learning baselines were trained with the same architecture, reward function, and other hyperparameters. We compare the performance of our method against baselines using the following metrics: (1) average reward, (2) fall recovery success rate, (3) torque applied: squared L2 norm of torques at every joint $∥\tau^{t}{∥}^{2}$, (4) smoothness which is derivative of torque $∥\tau^{t}-\tau^{t-1}{∥}^{2}$, and (5) ground impact: squared L2 norm of delta ground reaction forces at every foot $∥f^{t}-f^{t-1}{∥}^{2}$.

We compare the performance of RMA with several baselines in the simulation (Table 5). We sampled the training and test parameters according to Table 1. We resampled them in one set with a resampling probability of 0.004 and 0.01 per step for training and testing, respectively. The data in the report were averaged over eight random initializations and 1000 steps for each random initialization. Our method has the best performance, with only a slight degradation in performance compared to that of Expert. The changing environment leads to poor performance of the concurrent method, which adapts very slowly. This is because state estimation becomes unreliable in challenging environments, and AA cannot cope with this problem effectively. Note that the low performance without a state estimator means implicitly estimating is difficult, and there is no need to achieve superior performance.

## 5. Conclusions

This research aims to develop a highly dynamic fall recovery controller for legged robots. Unlike most solutions dedicated to narrow-skill tasks, we address this challenge with a unified end-to-end control architecture that generates adaptive motor skills and achieves a breadth of motor expertise. In particular, the policies learned using this architecture allow feedback strategies that respond quickly to changing situations. This is crucial for autonomous robots to react quickly in critical situations and contributes even more to mission success in real-world applications.

Although our current approach can generate adaptive control strategies, it is devoid of visual and haptic perception, which are critical for long-term motor planning [63] and dynamic manipulation [64]. To obtain more advanced motor intelligence in unstructured environments, future research needs to integrate visual cues and haptic perception to develop environmentally aware movement. While increasing the number of movement patterns, physical simulation training may pose some limitations. As the number of tasks increases, differences between the simulation and the real world may accumulate and become problematic. Based on the current results, future work will investigate learning algorithms that can safely refine motor skills on real hardware for more complex multi-modal tasks. Due to the complexity of high-dimensional problems, the introduction of more advanced neural network architectures such as Transformer [65] and Neural Architecture Search [66] is also a promising research direction.

## Figures and Tables

**Table 1:** Ranges of the randomized environmental parameter.

Term	Min	Max	Unit
Contact friction	0.4	1.25	-
Payload mass	−1.0	5.0	kg
Robot mass bias	85%	115%	-
Motor friction	0	0.05	Nm
Control frequency	60	70	Hz
Latency	0	40	ms
IMU bias	−0.05	0.05	rad
IMU noise (std)	0	0.05	rad
Motor encoder bias	−0.08	0.08	rad
Terrain height	−3	3	cm
External disturbance force	0	15	N
External disturbance torque	0	5	Nm
Gravity direction shift	0	15	deg

**Table 2:** Observations.

Data	Dimension	$o_{t}$	$c_{t}$	$x_{t}$	$e_{t}$
Desire velocity $v_{t}^{cmd}$	1		✓		
Desire direction $d_{t}^{cmd}$	1		✓		
Last action $a_{t-1}$	12	✓			
Joint angle $q_{t}$	12	✓			
Joint velocity ${\dot{q}}_{t}$	12	✓			
orientation $g_{t}^{\mathrm{ori}}$	3	✓			
Angular velocity $\omega_{t}^{\mathrm{ori}}$	3	✓			
Body velocity $v_{t}$	3			✓	
Binary foot contact indicator vector $f^{t}$	4			✓	
Relative position in the world frame $p_{t}$	3			✓	
Friction coefficient	1			✓	
Estimated body speed $v_{t}^{e}$	3				✓
Estimated foot contact state ${\mathrm{diag}}^{e}\left(f^{t}\right)$	4				✓

**Table 4:** The state estimator architecture.

Layer	State Estimator
input	44×100
1	Conv1d(44, 256, kernel size=5, stride=2, dilation=1)
2	Conv1d(256, 256, kernel size=5, stride=2, dilation=2)
3	Conv1d(256, 256, kernel size=5, stride=4, dilation=4)
4	Linear(256, 256)
6	Linear(256, 7)

**Table 5:** Simulation testing results.

Method	Reward	Success Rate (%)	Torque (Nm)	Smoothness (Nm)	Ground Impact (N)
Expert Policy	6.12	98.7	488.13	84.96	3.80
Our method	5.68	94.1	501.26	89.17	3.96
Without state estimator	5.15	81.9	572.22	92.21	3.91
Concurrent methods	5.29	75.7	522.83	101.32	4.30

## Data Availability

All data have been included in this paper.
